# Objective and Subjective Evaluations of the Effects of Different Types of Intubation Tube Applications on Voice Performance in the Early Postoperative Period

**DOI:** 10.7759/cureus.5835

**Published:** 2019-10-04

**Authors:** Selcuk Kayir, Guvenc Dogan, Dogan Atan

**Affiliations:** 1 Anesthesiology, Hitit University, Erol Olcok Training and Research Hospital, Corum, TUR; 2 Otorhinolaryngology, Lokman Hekim Ankara Hospital, Ankara, TUR

**Keywords:** voice, intubation, subjective, objective, postoperative

## Abstract

Objective

Changes in voice performance in the postoperative period due to trauma suffered during endotracheal intubation or edema occurring in the postoperative period are often observed. The present study aimed to evaluate the effects of different types of intubation tube applications on voice performance in the early postoperative period using objective and subjective voice analysis methods.

Materials and Methods

A total of 88 patients who underwent endotracheal intubation either using a cuffed endotracheal (*n* = 44) or spiral-embedded cuffed endotracheal (*n* = 44) tube were included in this study. An endotracheal tube of 7.5 mm was used for female patients and that of 8 mm was used for male patients. Preoperative acoustic voice analysis was performed, and fundamental frequency (F0), jitter%, and shimmer% values were recorded. In addition, the voice handicap index-30 (VHI-30) questionnaire was completed by the patients for subjective evaluation. The same procedure was repeated in the first 48 hours postoperatively. The preoperative and postoperative data were statistically compared. In addition, the effect of the type of endotracheal intubation tube on acoustic voice analysis parameters and VHI-30 scores was statistically evaluated.

Results

In the early postoperative period, a significant decrease in the F0 value and a significant increase in jitter% and shimmer% values were detected. The VHI-30 score was also found to be significantly higher in the early postoperative period than in the preoperative period. The effects of both the intubation tubes on voice performance were found to be similar.

Conclusion

Objective and subjective evaluations revealed that voice performance was declined in the early postoperative period after orotracheal intubation.

## Introduction

Anatomical structures, such as the oral cavity, oropharynx, nasal cavity, and laryngeal structures, contribute markedly to the production of voice. The structural and/or anatomical defects in the upper airway may affect the resonance of voice and articulation and may lead to a decline in voice performance [1]. In the literature, it has been reported that surgical procedures in the upper respiratory tract, acute and/or chronic infections, vocal cord paralysis, and vocal cord pathologies, as well as surgical interventions to this region, affect voice performance [2-5].

Currently, endotracheal intubation is a commonly used method in patients undergoing surgery under general anesthesia. However, complaints, such as sore throat, hoarseness, and difficulty in swallowing, have been reported by patients in the early postoperative period after this procedure. The main cause of these symptoms is trauma to the vocal cords during intubation. Voice performance can be affected during early postoperative period due to the injury of the vocal cords.

Studies evaluating voice performance after endotracheal intubation have been reported in the literature [6-7]. In these studies, voice performance was evaluated by acoustic voice analysis, which is an objective evaluation. In our study, voice performance was evaluated using the voice handicap index-30 (VHI-30) questionnaire, which is a subjective evaluation method, in addition to the acoustic voice analysis. To the best of our knowledge, we believe that our study is the first to report voice performance using subjective evaluations.

## Materials and methods

This study was conducted between January 2019 and March 2019 at the Hitit University Erol Olçok Education and Research Hospital. This study was approved by the Ethics Committee of Hitit University Medical Faculty, and informed consent was obtained from the patients. This study included 88 patients aged 18-80 years, conforming to the ASA I-II class according to the classification of the American Society of Anesthesiologists (ASA), who were scheduled to undergo surgery that will not last more than 120 minutes with the patients in the supine position, except an airway or laparoscopic surgery. The examinations of the nasopharynx and larynx of the patients were performed with the preoperative and postoperative oral cavity, oropharynx, and flexible fiberoptic examinations.

Patients with a lung disease, those having obesity (BMI >35 kg.m^-2^), pregnant patients, those with a history of gastroesophageal reflux or suspected of having difficult airway (mouth opening <2.5 cm, Mallampati score >2, sternomental distance <12.5 cm, thyromental distance <6 cm, neck circumference >40 cm), those with a high risk of aspiration pneumonia, those on inhaled steroids, those with airway obstruction due to upper airway pathology, and those with active infection were excluded from the study. The patients were randomly divided into four groups according to the closed envelope method: Group ET-7.5, a 7.5-mm cuffed endotracheal tube (ET; *n* = 22); Group ET-8, an 8-mm cuffed ET (*n* = 22); Group SET-7.5, a 7.5-mm spiral-embedded cuffed ET (*n* = 22); and Group SET-8, an 8-mm spiral-embedded cuffed ET (*n* = 22).

During the operation, premedication was not performed in the patients according to the general anesthesia protocol. In the operating room, after monitoring the heart rate, non-invasive arterial blood pressure, and SpO_2_ (partial oxygen pressure) in the patients, anesthesia was induced with propofol 3 mg.kg^−1^, fentanyl 2 mcg.kg^-1^, and rocuronium bromide 0.5 mg.kg^−1^. Patients were ventilated with 100% O_2_ for three minutes at a flow rate of 6 lt.min^-1^, and orotracheal intubation was performed by an expert anesthetist in a single attempt using an ET that conforms to the patient’s age and body weight. The cuff of the intubation tube was inflated with atmospheric air until the leakage sound ceased. The visualization of the capnograph wave and bilateral chest expansion during manual ventilation were considered as the indicators of effective ventilation. Anesthesia was maintained with 1% to 2% sevoflurane in 50%/50% O_2_ air mixture and remifentanil infusion by controlled mechanical ventilation to achieve a tidal volume of 6-8 mL.kg^-1^ and respiratory rate of 12 breaths per minute using a Datex Ohmeda S/5 Avance anesthesia machine in all patients. In all the groups, neuromuscular blocks were reversed, and the ET removal after spontaneous respiration was achieved.

Acoustic voice analysis was performed on the patients preoperatively and in the first 48 hours postoperatively. In the acoustic voice analysis, F0, jitter%, and shimmer% were examined. In addition, the VHI-30 questionnaire was completed under the supervision of a physician for the subjective evaluation of voice quality. The preoperative and postoperative values of F0, jitter%, shimmer%, and VHI-30 scores between the groups were statistically compared.

Preoperative and postoperative acoustic voice analysis parameters and VHI-30 scores of the ET group (Group ET-7.5 and Group ET-8) and SET group (Group SET-7.5 and Group SET-8) were statistically compared.

The effect of 7.5- and 8-mm cuffed intubation tubes on preoperative and postoperative acoustic voice analysis parameters, and VHI-30 scores were statistically evaluated.

Acoustic voice analysis

For voice recordings, an Audio-Technica AT 2005 model dynamic microphone (Audio-Technica productions, Western Hemisphere, USA) was used at a distance of 5 cm from the mouth in a room that is far from the hospital crowd where environmental noise is minimal. Recordings of all patients were performed as a mono sound recording at a sampling rate of 44,100 Hz and in a 16-bit sampling format. For acoustic voice analysis, the patients were asked to utter the vowel “a” for five seconds three times. The average of three utterances was recorded. In the acoustic voice analysis, F0, jitter%, and shimmer% were evaluated. All acoustic evaluations were performed online using Windows 7 operating system via Praat (Paul Boersma, 2001. Version 6.017, http://www.praat.org/) program. Praat is an easy-to-use non-invasive computer program that can measure various aspects of sound. Spectrograms in Praat were used to evaluate the format frequencies.

Voice handicap index

The patients completed the “voice handicap index” questionnaire before and after the surgery for the subjective evaluation of voice [8]. According to the responses on this scale, the degree of physical, social, and functional handicaps of the patients’ voice in daily life was determined. Each item is scored on a five-point scale (0-4). The total score is between 0 and 120. A high score indicates severe subjective voice impairment.

Statistical analysis

Statistics were performed using SPSS (version 21.0, Statistical Package for Social Sciences). Descriptive statistics were presented as the mean ± standard deviation (SD). Categorical variables were presented as the percentage. Distribution of data was evaluated with Shapiro-Wilks test. Chi-square test, Fisher's exact test, and Students' *t*-test were used to compare demographic data of the groups. Paired *t*-test was used to analyze normally distributed continuous variables, and the Wilcoxon signed-rank test was used for normally distributed ones for pre-post operational data. Statistical significance was accepted for values of *p *< 0.05.

## Results

Among the 88 patients included in the study, 44 were in the ET groups and 44 were in the SET group. While 23 male and 21 female patients were included in the ET group, 21 male and 23 female patients were included in the SET group. The mean age was 42.64 and 42.05 years in the ET and SET groups. Both groups were found to be statistically compatible when matched for sex and age (Table 1).

**Table 1:** Demographic data of ET group and SET group ^a^ Chi-square test, ^b^ Fisher's exact test, ^c^ Student's *t*-test ET, cuffed endotracheal tube; SET, spiral-embedded cuffed endotracheal tube; SD, standard deviation; M, male; F, female

	ET group (n = 44) Mean ± SD	SET group (n = 44) Mean ± SD	P-value
Gender	23 m/ 21 f	21 m/ 23 f	0.670 ^a^
Age	42.64 ± 13.52	42.05 ± 12.81	0.834 ^c^
Smoking	16 (% 36.3)	13 (% 29.5)	0.496^ a^
Use of alcohol	2 (%4.5)	0	0.494 ^b^

The acoustic voice analysis parameters and VHI-30 scores of the ET and SET groups recorded preoperatively and in the first 48 hours postoperatively were compared using statistical methods. In the ET group, the mean preoperative F0 value was 168.74 Hz, whereas the mean postoperative F0 value was 157.1 Hz. In the SET group, the mean preoperative F0 value was 161.55 Hz, whereas the mean postoperative F0 value was 157.59 Hz. In both the groups, a significant decrease in the F0 value in the early postoperative period (*p *= 0.003, *p* = 0.042; Table 2) was noted.

In the ET group, the mean preoperative jitter% value was 0.46 and the mean postoperative jitter% value was 0.8. In the SET group, the mean preoperative jitter% value was 0.43, whereas the mean preoperative jitter % value was 1.01. In both the groups, a significant increase was noted in the jitter% value in the early postoperative period (*p *< 0.001, *p *= 0.001; Table 2).

In the ET group, the mean preoperative shimmer% value was 3.03 and the mean postoperative shimmer% value was 3.71. In the SET group, the mean preoperative shimmer% value was 3.35 and the mean postoperative shimmer% value was 3.97. In both the groups, a significant increase was noted in the shimmer% value in the early postoperative period (*p *= 0.027, *p* = 0.048; Table 2).

The mean pre- and postoperative scores on the VHI-30 questionnaire that was administered for the subjective evaluation of voice were 4.5 and 7.29 in the ET group and 3.61 and 6.81 in the SET group, respectively. The changes in the scores from baseline were found to be significant (*p *< 0.001 for the ET group and *p *< 0.001 for the SET group; Table 2). It was observed that voice was affected in both objective and subjective evaluation.

**Table 2:** Preoperative and postoperative; F0, jitter%, shimmer% and VHI-30 values and statistical analysis for ET and SET groups ET, cuffed endotracheal tube; SET, spiral-embedded cuffed endotracheal tube; SD, standard deviation; F0, fundamental frequency; VHI, voice handicap index

	ET group (n = 44) Preoperative Mean ± SD	ET group (n = 44) Postoperative Mean ± SD	P-value	SET group (n = 44) Preoperative Mean ± SD	SET group (n = 44) Postoperative Mean ± SD	P-value
F0	168.74 ± 51.99	157.10 ± 46.95	0.003	161.55 ± 46.07	157.59 ± 50.5	0.042
Jitter%	0.46 ± 0.89	0.8 ± 1.1	<0.001	0.43 ± 0.49	1.01 ± 1.73	0.001
Shimmer%	3.03 ± 2.32	3.71 ± 2.59	0.027	3.35 ± 1.86	3.97 ± 2.52	0.048
VHI-30	4.5 ± 4.31	7.29 ± 5.15	<0.001	3.61 ± 4.41	6.81 ± 6.11	<0.001

In the evaluation of female patients with a 7.5-mm endotracheal intubation tube and in the evaluation of male patients with an 8-mm endotracheal intubation tube, a significant decrease in the F0 values in the early postoperative period and a significant increase in the jitter% and shimmer% values and VHI-30 parameters were noted in both the groups (Table 3). 

**Table 3:** 7.5 and 8.0 ET groups; F0, jitter%, shimmer%, and VHI-30 values and statistical analysis ET, endotracheal tube; SD, standard deviation; F0: fundamental frequency; VHI, voice handicap index

	7.5 ET (n = 44) Preoperative Mean ± SD	7.5 ET (n = 44) Postoperative Mean ± SD	P-value	8.0 ET (n = 44) Preoperative Mean ± SD	8.0 ET (n = 44) Postoperative Mean ± SD	P-value
F0	200.26 ± 40.98	157.10 ± 46.95	0.001	130.04 ± 25.51	127.08 ± 27.79	0.024
Jitter%	0.44 ± 0.52	0.94 ± 1.61	<0.001	0.45 ± 0.87	0.87 ± 1.26	<0.001
Shimmer%	3.24 ± 2.1	4.04 ± 2.4	0.013	3.14 ± 2.11	3.64 ± 2.69	0.029
VHI-30	3.95 ± 4.91	7.09 ± 5.53	<0.001	4.15 ± 3.78	7.02 ± 5.78	<0.001

## Discussion

In our study, objective and subjective evaluations revealed that endotracheal intubation causes a decline in voice performance in the early postoperative period; however, this decline was not associated with the type and size of intubation tubes.

The anatomical structures of the upper airway contribute markedly to the production of voice, but the most important anatomical structure is the larynx. The character of voice is shaped in the vocal cords; therefore, a problem at any location in the upper airway can affect voice performance [9]. Benign lesions of the larynx (polyp, nodule, cyst, and Reinke’s edema), malignant tumors, infections, laryngopharyngeal reflux, unilateral or bilateral vocal cord paralysis, and traumatic lesions affect voice quality adversely [5,10]. In our study, patients with an infection, obstruction, or benign-malignant diseases in any part of the upper respiratory tract were excluded from the study.

There may be changes in the postoperative voice performance due to trauma occurring during orotracheal intubation or edema occurring thereafter in the larynx. A study based on a large series of patients conducted by Sorensen et al. reported a significant increase in jitter% and shimmer% values in the acoustic voice analysis performed 12-24 hours after surgery [6]. In another study evaluating the postoperative acoustic voice analysis of patients who underwent thyroidectomy and parathyroidectomy, patients with nerve damage and/or injury were excluded, and a significant deterioration in the F0 and shimmer% values was detected in the first 24 hours postoperatively [11]. In our study, acoustic voice analysis performed in the first 48 hours postoperatively revealed significant deteriorations in the F0, jitter%, and shimmer% values compared with those values in the preoperative period.

The type and size of intubation tubes also influence laryngeal trauma and edema occurring during and after intubation. Sorensen, et al. examined the postoperative effects of an Endoflex and normal intubation tube on the larynx and voice performance [7]. Two groups were found to be similar in terms of hoarseness and vocal cord injury. In the same study, a lower increase was noted in shimmer % values in the Endoflex group and this was explained by the fact that the Endoflex tube caused less laryngeal mobility [7]. In our study, it was found that normal intubation tube and spiral intubation tube had negative effects on the sound performance similar to the first 48 hours postoperatively, indicating that both intubation tubes have no superiority to each other in the early postoperative period.

To the best of our knowledge, no study in the literature has used subjective evaluations to investigate the effect of orotracheal intubation on voice performance. In our study, the VHI-30 questionnaire was used for subjective evaluation in addition to acoustic voice analysis. The VHI-30 score was found to be significantly higher, regardless of the type and size of the tube, in the first 48 hours postoperatively. In this respect, voice performance was found to be subjectively deteriorated. We believe that our study is the first in the literature to report such subjective evaluations.

The present study had some limitations. The effect of intubation tubes of different sizes in male and female patients on voice performance has not been investigated. The period when the voice that was affected in the first 48 hours recovered was not evaluated. In addition, the same procedures and medical treatment algorithms were used for general anesthesia in our study. The effect of different medical treatment algorithms on voice performance can also be investigated. Future studies investigating these areas and involving large series of patients are warranted to contribute to the literature.

## Conclusions

In our study, it was determined that orotracheal intubation impaired voice performance in the first 48 hours postoperatively. However, this effect was not associated with the type and size of intubation tubes. In addition, in our study, the sound performance was measured as subjective. In this respect, voice performance was found to be subjectively deteriorated.
